# Rethinking risk in Crohn’s surgery: age at onset fails to predict surgical outcomes after ileocecal resection, insights from a tertiary referral center

**DOI:** 10.1007/s10151-026-03304-w

**Published:** 2026-04-22

**Authors:** T. Violante, S. Cardelli, F. Flamini, G. Calini, M. Novelli, M. Rottoli

**Affiliations:** 1 Surgery of the Alimentary Tract, IRCCS Azienda Ospedaliero-Universitaria Di Bologna, Bologna, Italy; 2 Department of Medical and Surgical Sciences, Alma Mater Studiorum - University of Bologna, Via Massarenti 9, 40138 Bologna, Italy; 3 Division of Colon and Rectal Surgery, Mayo Clinic, Rochester, MN USA; 4 Department of Statistical Sciences, Alma Mater Studiorum - University of Bologna, Bologna, Italy

**Keywords:** Crohn’s disease, Ileocecal resection, Surgical outcomes, IBD, Montreal classification, Surgical classification

## Abstract

**Background:**

Early age at diagnosis in Crohn’s disease is linked to aggressive phenotypes, yet evidence regarding its impact on surgical risk remains inconsistent. This study aimed to elucidate the relationship between age at diagnosis, utilizing Vienna and Montreal classifications, and surgical prognosis in patients undergoing ileocecal resection.

**Methods:**

A retrospective analysis of a prospective database identified 810 patients undergoing ileocecal resection between 2014 and 2022. Patients were stratified by Vienna (< 40 versus > 40 years) and Montreal (A1, A2, A3) classifications. Primary end points included 30-day overall complications, serious complications, reoperation and readmission. Statistical analysis employed multivariable regression, propensity score matching, G-computation and Random Forest models to adjust for confounders.

**Results:**

Baseline characteristics differed significantly: younger patients exhibited more penetrating disease and biologic exposure, while older patients had higher ASA scores and comorbidities. After robust adjustment, the Vienna and Montreal age classification showed no significant association with postoperative complications, serious complications, reoperation or readmission. Random Forest analysis consistently identified ASA score and comorbidities, rather than age at onset, as the dominant predictors of surgical outcomes.

**Conclusions:**

Age at diagnosis does not independently predict short-term surgical outcomes after ileocecal resection. Postoperative morbidity is driven primarily by general health markers, such as ASA score, rather than disease onset timing. These findings highlight the need for validated disease-specific risk scores.

**Supplementary Information:**

The online version contains supplementary material available at 10.1007/s10151-026-03304-w.

## Introduction

Crohn’s disease, first reported by Crohn, Ginzburg and Oppenheimer in 1932 [1] has seen a considerable growth in incidence within developed countries such as North America and Northern/Western Europe [2].

Despite advances in medical therapy, surgery remains a cornerstone of Crohn’s disease management owing to the high prevalence of stricturing, penetrating complications and refractory cases [3–5]. The complexity of care demands lifelong, individualized strategies that integrate disease phenotype, activity, treatment response and patient-specific risk factors, with the overarching goals of achieving durable remission, minimizing complications, improving quality of life and optimizing surgical outcomes [6–8].

Accurate disease classification is essential for guiding both medical and surgical decision-making. The Vienna and Montreal classification systems have been widely adopted for their ability to stratify patient risk and inform prognosis [9, 10].

Both schemes associate early age at diagnosis with a more extensive and aggressive phenotype, increased need for advanced therapies, and a greater likelihood of early surgical intervention. However, the evidence does not consistently show higher overall surgical rates or poorer postoperative outcomes in early-onset versus late-onset cases [11–13].

Notably, data specifically addressing the influence of age at diagnosis and disease onset timing on surgical outcomes remain limited. This study aims to address this gap and elucidate the relationship between age at diagnosis and surgical prognosis in Crohn’s disease.

## Methods

After International Review Board (IRB) approval, we retrospectively reviewed an institutional prospective database of patients with Crohn’s disease who underwent ileocecal resection. The final cohort included patients with a confirmed diagnosis of Crohn’s disease who underwent an ileocecal resection between January 2014 and December 2022. The cohort included patients undergoing elective and emergency ileocecal resections, although emergency presentations were rare (< 1%). Both primary (de novo) and redo resections were included in the analysis.

Patients were classified according to age at diagnosis following both the Vienna and Montreal systems. The Vienna classification divided patients into two groups: younger than 40 years and 40 years or older, while the Montreal classification stratified them into three categories: A1 (< 16 years), A2 (17–40 years), and A3 (> 40 years).

Baseline demographic and clinical variables included age at diagnosis and at surgery, sex, body mass index (BMI), comorbidities (hypertension, cardiovascular events, diabetes, COPD, kidney insufficiency, bleeding disorders, thyroid disorders), smoking status, disease duration, prior biologic therapy, steroid use at surgery, preoperative total parenteral nutrition (TPN), Crohn’s disease behaviour (penetrating, stricturing or mixed type), years of disease evolution and ASA score. Surgical variables recorded were operative time, surgical approach (laparoscopic or open), conversion rate to open surgery in laparoscopic procedures, stoma creation and additional procedures. Postoperative outcomes included 30-day morbidity such as ileus (defined as the need for a nasogastric tube or inability to resume oral intake for more than 48 h), intra-abdominal collections, surgical site infections, small bowel obstruction, anastomotic leak and pneumonia. Serious complications were graded according to the Clavien–Dindo classification (> III). Reoperation and readmission rates within 30 days, length of hospital stay and need for postoperative TPN were also analysed.

To determine the effect of age categories for both the Vienna and the Montreal classification on surgical outcomes, the following primary variables were chosen as primary end points: operative time, 30-day postoperative complications, 30-day postoperative serious complications, 30-day readmission and reoperation and length of hospital stay.

### Statistical analysis

Statistical analysis was performed using R software (Version 4.5.1). A *p*-value of < 0.05 was considered statistically significant. Descriptive statistics were used to summarize patient demographics and clinical characteristics; continuous variables were reported as median and interquartile range (IQR), while categorical variables were presented as counts (*n*) and percentages (%). Categorical variables were compared using the Chi-squared test or Fisher’s exact test as appropriate, while continuous variables were compared using the Mann–Whitney *U* or Kruskal–Wallis tests.

To compare outcomes between the age categories using both the Vienna and Montreal classification, four distinct analytical approaches were employed to ensure robustness and control for confounding. First, unadjusted (Raw) comparisons were made. Second, multivariable models (Multi) were constructed, using logistic regression for binary outcomes and linear regression for continuous outcomes, utilizing robust standard errors. Third, Propensity Score Matching (PSM), using nearest neighbour matching, was performed to create balanced cohorts [14]. Fourth, G-computation (g-comp) was used as an additional causal inference method, with confidence intervals derived from bootstrap-based techniques [15]. All adjusted analyses (Multi, PSM, g-comp) controlled for the following covariates: body mass index (BMI), age at surgery, gender, presence of comorbidities, history of previous abdominal surgery, steroid use at the time of surgery, preoperative biologic use, duration of disease (years), disease behaviour and ASA score. Outcomes are reported as Odds Ratios (OR) for binary variables and beta coefficients (*β*) for continuous variables, both with 95% confidence intervals (95% CI).

In addition, to explore variable importance and predictive power in relation to postoperative outcomes, a Random Forest machine learning analysis was conducted [16]. Variable importance was assessed using Mean Decrease in Accuracy. Mean Decrease in Accuracy estimates the impact on model accuracy when a variable’s values are permuted, with greater decreases indicating higher importance.

## Results

A total of 810 patients undergoing ileocecal resection were included. According to the Vienna classification (Table 1), 590 patients (72.8%) were diagnosed before age 40 and 220 (27.2%) after age 40. In the unadjusted analysis, baseline characteristics differed significantly between groups. Patients aged > 40 had higher rates of comorbidities (56.4% versus 23.9%, *p* < 0.001), higher ASA scores (ASA > 3: 27.7% versus 10.1%, *p* < 0.001) and a higher median BMI (22.3 versus 20.6 kg/m^2^, *p* < 0.001). They were also more frequently current or former smokers (*p* < 0.0001) and more commonly presented with stricturing disease (*p* = 0.01), whereas penetrating disease predominated in the younger group (*p* = 0.01). Younger patients had a longer disease duration (median 7 versus 4 years, *p* < 0.0001) and more frequent exposure to biologic therapy before surgery (40.7% versus 30.0%, *p* = 0.006).

**Table 1:** Surgical outcomes across Vienna age categories

Variables	Age < 40 (*N* = 590)	Age > 40 (*N* = 220)	*p*-value
Gender (male)	304 (51.5)	121 (55.0)	0.8^1^
Age at diagnosis (years), median ± IQR	23 ± 11	49 ± 14	** < 0.001**^**3**^
Age at surgery (years), median ± IQR	31 ± 15	56 ± 14	** < 0.001**^**3**^
Body mass index (Kg/m^2^), median ± IQR	20.6 ± 4.9	22.3 ± 4.3	** < 0.001**^**3**^
*ASA score* 1 2 3	30 (5.1) 500 (84.8) 60 (10.1)	0 (0) 159 (72.3) 61 (27.7)	** < 0.001**^2^
*Comorbidities*	141 (23.9)	124 (56.4)	** < 0.001**^2^
Smoking Status Non-smoker Past-smoker Active smoker	346 (58.6) 160 (27.1) 84 (14.3)	94 (42.7) 73 (33.2) 53 (24.1)	** < 0.001**^2^
*Disease behaviour* Stricturing Penetrating Stricturing and penetrating	263 (44.6) 58 (8.5) 277 (46.9)	128 (58.2) 7 (3.2) 85 (38.6)	**0.01**^**2**^ **0.01**^**2**^ 0.09^2^
Years of disease, median ± IQR	7 ± 10	4 ± 8	** < 0.001**^**3**^
Steroids at surgery	162 (27.5)	51 (23.2)	0.2^1^
Previous use of biologics	240 (40.7)	66 (30)	**0.006**^**1**^
Preoperative TPN	48 (8.1)	24 (10.9)	0.2^1^
*Surgical approach* Laparoscopic Open	394 (66.8) 196 (33.2)	141 (64.1) 79 (35.9)	0.4^1^
Additional procedures	269 (45.6)	77 (35)	**0.007**^**1**^
Operative time (min), median ± IQR	172 ± 75	178 ± 73	0.5^3^
Conversion to open	47 (11.9)	30 (21.3)	**0.01**^**1**^
Stoma construction	68 (11.5)	21 (9.6)	0.4^1^
Intraoperative transfusion	29 (4.9)	19 (8.6)	**0.04**^**1**^
Postoperative TPN	64 (10.9)	30 (13.6)	0.3^1^
Length of stay (days), median ± IQR	7 ± 2	8 ± 3	** < 0.001**^**3**^
30-day postoperative complication	213 (36.1)	94 (42.7)	0.09^1^
30-day serious complication	48 (8.1)	26 (11.8)	0.1^1^
30-day readmission	33 (5.6)	8 (3.6)	0.3^2^
30-day postoperative reoperation	28 (4.8)	14 (6.4)	0.4^1^

Values are *n* (%) unless otherwise indicated; ^1^Chi squared; ^2^Fischer’s exact test; ^3^ Wilcoxon rank sum, Mann–Whitney *U* test. Bold numbers indicate statistically significant results (*p* < 0.05)

Surgical characteristics also varied by age. Patients > 40 years old had higher rates of conversion to open surgery (21.3% versus 11.9%, *p* = 0.01) and intraoperative transfusion (8.6% versus 4.9%, *p* = 0.04), as well as a longer median postoperative stay (8 versus 7 days, *p* < 0.001). Younger patients underwent significantly more additional procedures (45.6% versus 35.0%, *p* = 0.007). These procedures primarily consisted of strictureplasties (e.g., Heineke–Mikulicz, Finney) and limited segmental small bowel resections required for multifocal disease. There were no significant unadjusted differences between the two Vienna age groups in 30-day overall complications, serious complications, readmission or reoperation.

Using the Montreal classification (Table 2), 101 patients (12.5%) were categorized as A1 (< 16 years), 498 (61.5%) as A2 (17–40 years), and 211 (26.0%) as A3 (> 40 years). Significant differences were observed across the three groups in BMI (*p* = 0.0001), ASA score (*p* < 0.0001), comorbidity status (*p* < 0.0001) and smoking history (*p* < 0.0001). Disease behaviour also varied significantly (*p* < 0.02–0.0001), as did disease duration (*p* = 0.0001) and prior biologic therapy (*p* = 0.0001).

**Table 2:** Surgical outcomes across Montreal age categories

Variables	A1 < 16 years old (*N* = 101)	A2 (between 17 and 40) (*N* = 498)	A3 > 40 years old (*N* = 211)	*p*-value
Gender (male)	47 (46.5)	263 (52.8)	115 (54.5)	0.4^1^
Age at diagnosis (years), median ± IQR	14 ± 5	25 ± 10	50 ± 15	** < 0.0001**^**2**^
Age at surgery (years), median ± IQR	20 ± 8	34 ± 15	57 ± 14	** < 0.0001**^**2**^
Body mass index (Kg/m^2^), median ± IQR	19.6 ± 5.5	20.8 ± 5.3	22.3 ± 4.3	**0.0001**^**2**^
*ASA score* 1 2 3	12 (11.9) 78 (77.2) 11 (10.9)	18 (3.6) 430 (86.4) 50 (10.4)	0 (0) 151 (71.6) 60 (28.4)	** < 0.0001**^**1**^
*Comorbidities*	20 (19.8)	122 (24.5)	123 (58.3)	** < 0.0001**^**1**^
Smoking status, Non-smoker Past-smoker Active smoker	83 (82.2) 12 (11.9) 6 (5.9)	266 (53.4) 152 (30.5) 80 (16.1)	91 (43.1) 69 (32.7) 51 (24.2)	** < 0.0001**^**1**^
*Disease behaviour* Stricturing Penetrating Stricturing and penetrating	53 (52.5) 12 (11.9) 12 (11.9)	214 (43.0) 38 (7.6) 38 (7.6)	124 (58.8) 7 (3.3) 7 (3.2)	** < 0.0001**^**1**^ **0.01**^**1**^ **0.02**^**1**^
Years of disease, median ± IQR	7 (8)	8 (11)	4 (7)	0.0012
Steroids at surgery	38 (37.6)	193 (38.8)	66 (31.3)	0.1^1^
Previous use of biologics	63 (62.4)	181 (36.5)	62 (29.4)	**0.0001**^**2**^
Preoperative TPN	9 (8.9)	41 (8.2)	22 (10.4)	0.6^1^
*Surgical approach* Laparoscopic Open	71 (70.3) 30 (29.7)	328 (65.9) 170 (44.1)	136 (64.5) 75 (35.5)	0.6^1^
Additional procedures	49 (48.5)	223 (44.8)	74 (35.1)	**0.03**^**1**^
Operative time (min), median ± IQR	165 ± 65	175 ± 80	175 ± 70	0.2^2^
Conversion to open	8 (11.3)	41 (12.5)	28 (20.6)	0.06^1^
Stoma construction	7 (6.9)	62 (12.5)	20 (9.5)	0.2^1^
Intraoperative transfusion	8 (7.9)	22 (4.2)	18 (8.5)	0.07^1^
Postoperative TPN	19 (18.8)	45 (9.0)	30 (14.2)	**0.008**^**1**^
Length of stay (days), median ± IQR	7 ± 3	7 ± 2	8 ± 3	**0.0001**^**2**^
30-day postoperative complication	43 (42.6)	174 (34.9)	90 (42.7)	0.09^1^
30-day serious complication	15 (14.9)	35 (7.0)	24 (11.4)	**0.01**^**1**^
30-day readmission	7 (6.9)	26 (5.2)	8 (3.8)	0.5^1^
30-day postoperative reoperation	12 (11.9)	17 (3.4)	13 (6.2)	**0.002**^**1**^

Values are *n* (%) unless otherwise indicated; ^1^Chi squared; ^2^Kruskall–Wallis test. Bold numbers indicate statistically significant results (*p* < 0.05)

Patients in the A1 and A2 groups underwent more additional procedures (*p* = 0.03), while postoperative total parenteral nutrition (TPN) was more frequently required by A1 and A3 patients compared with A2 (*p* = 0.008). Hospital stay differed significantly, with A3 patients remaining hospitalized longest. The A1 and A3 groups had higher rates of serious postoperative complications than A2 (14.9% and 11.4% versus 7.0%, *p* = 0.01), and the A1 group also showed the highest reoperation rate (11.9%, *p* = 0.002). A1 patients had the greatest exposure to biologics before surgery (62.4% versus 36.5% for A2 and 29.4% for A3, *p* < 0.0001) and the highest postoperative TPN use (18.8% versus 9.0% and 14.2%, *p* = 0.008). Across Montreal age strata, increasing age correlated with higher ASA scores, greater comorbidity burden and longer hospital stays (all *p* < 0.001).

After adjustment for confounders including gender, BMI, ASA class, comorbidities, disease duration, steroid and biologic use, surgical approach and additional procedures using multivariable regression, propensity score matching (PSM) and G-computation (Table 3), the Vienna classification (age > 40 versus < 40) was not significantly associated with 30-day postoperative complications (e.g., PSM OR: 1.185, *p* = 0.384), serious complications (PSM OR: 1.504, *p* = 0.208), reoperation (PSM OR: 1.593, *p* = 0.290) or readmission (PSM OR: 0.654, *p* = 0.365). The only finding consistently significant across adjusted models was a longer hospital stay in the > 40 group (β = 1.9–2.1 days, *p* < 0.002).

**Table 3:** Adjusted associations between age at diagnosis (Vienna Classification) and surgical outcomes (propensity score matched analysis)

Outcome	Vienna Classification(< 40 versus > 40 years)	Montreal Classification(Comparison versus A1 < 17 years)
	Effect size (95% CI)	
Operative time (min)	β: 5.15 (−6.79 to 17.09) *p* = 0.397	**A2 versus A1: β: 10.25 (0.32–20.17), p = 0.043** **A3 versus A1: β: 10.83 (0.06–21.61),** ***p* = 0.049**
30-day Complications	OR: 1.18 (0.81–1.74) *p* = 0.384	A2 versus A1: OR: 0.79 (0.56–1.11), *p* = 0.173 A3 versus A1: OR: 0.91 (0.63–1.30), *p* = 0.588
Serious complications	OR: 1.50 (0.80–2.88) *p* = 0.208	A2 versus A1: OR: 0.65 (0.31–1.34), *p* = 0.244 A3 versus A1: OR: 0.78 (0.37–1.63), *p* = 0.510
30-day readmission	OR: 0.65 (0.25 – 1.62) *p* = 0.365	A2 versus A1: OR: 1.05 (0.45–2.45), *p* = 0.902 A3 versus A1: OR: 0.68 (0.23–2.03), *p* = 0.490
30-day reoperation	OR: 1.59 (0.68–3.91) *p* = 0.290	A2 versus A1: OR: 0.51 (0.19–1.36), *p* = 0.180 A3 versus A1: OR: 0.44 (0.13–1.52), *p* = 0.200
Length of stay (days)	**β: 2.15 (0.83–3.47) *p* = 0.002**	A2 versus A1: β: -0.21 (−1.22 to 0.81), *p* = 0.689 A3 versus A1: β: 0.69 (−0.48 to 1.87), *p* = 0.246

Results represent the propensity score matched (PSM) analysis. Vienna: Comparison reference is age > 40. Montreal: Comparison reference is Group A1 (< 17 years). *OR*   Odds Ratio; *beta*   Beta coefficient; *CI*   Confidence Interval. Bold values indicate statistical significance (*p* < 0.05)

In adjusted Montreal analyses using the A1 group (< 16 years) as reference, the A2 group (17–40 years) had significantly lower odds of 30-day serious complications (PSM OR: 0.548, 95% CI [0.308–0.956], *p* = 0.036) and reoperation (PSM OR: 0.361, 95% CI [0.168–0.732], *p* = 0.006). Similarly, the A3 group (> 40 years) showed reduced odds of reoperation in PSM (OR: 0.482, 95% CI [0.227–0.969], *p* = 0.047). Both A2 and A3 groups had longer operative times compared with A1 (β = 10–14 min, *p* < 0.05), but no significant differences in overall 30-day complications or readmission were observed between groups. The complete results of the unadjusted, multivariable and sensitivity analyses for both Vienna and Montreal classifications are provided in Supplementary Tables 1 and 2.

To further assess the relative importance of clinical and surgical factors across age classifications, Random Forest models (Supplementary Fig. 1–10) were independently constructed for the Montreal and Vienna groupings. In the Montreal-based model, ASA score, surgical approach and comorbidities emerged as the strongest predictors of postoperative complications. Similar findings were observed with the Vienna-based model, where ASA score, comorbidities dominated predictive rankings. These consistent patterns across both classification systems suggest that, while age groups describe distinct clinical phenotypes, age at diagnosis itself is not a principal determinant of surgical risk. The Random Forest analyses traditional methods by capturing nonlinear interactions, providing a more nuanced understanding of factors influencing postoperative outcomes in Crohn’s disease surgery, these analyses were performed also for serious complications, operative time and length of stay.

## Discussion

This large single-centre study demonstrates that age at diagnosis, classified by either the Vienna or Montreal systems, does not independently predict adverse surgical outcomes for patients undergoing ileocecal resection for Crohn’s disease. This conclusion held true even after applying robust statistical adjustments, including multivariable regression, propensity score matching and G-computation, to control for confounding variables. Specifically, our analysis found no significant association between a patient’s age at diagnosis and key postoperative endpoints, including overall complications, serious complications, reoperation rates or readmission rates. Although older age was associated with a longer hospital stay, the core measures of surgical morbidity were not driven by the timing of disease onset.

It is widely described that early-onset disease is associated with higher exposure to immunosuppressants, biological therapy and a higher risk of surgery, a trend reflected in our own early-onset groups. However, as this study demonstrates, early-onset CD did not translate to worse postoperative outcomes in terms of complications, readmissions and reoperations [17–20]. This aligns with recent studies and guidelines reporting that postoperative risk is more closely linked to factors such as malnutrition, preoperative sepsis, frailty and emergency surgery, rather than age at diagnosis or disease phenotype alone. Our findings also resonate with our random forest analysis, which showed that general health status (ASA, comorbidities, BMI) and the type of surgery (surgical approach, additional procedures, conversion to open surgery) were more important in determining the main short-term postoperative outcomes [9, 21, 22].

Our analysis confirms that modifiable factors such as ASA score and comorbidities, rather than the age of disease onset, are the primary drivers of surgical risk. This suggests that perioperative management should prioritize preoperative optimization and prehabilitation programs aimed at nutritional and physical optimization, as these interventions may offer greater benefit than risk stratification based purely on disease demographics.

The findings of this study underscore a critical, unmet need: the development of standardized, validated risk scores specifically for predicting surgical outcomes in Crohn’s disease. While the Vienna and Montreal classification systems are valuable for categorizing disease phenotype and guiding medical management, our analysis reveals their inadequacy as reliable predictors of perioperative morbidity. This observation aligns with mounting evidence that traditional classification schemes fail to capture the multifactorial determinants of surgical risk [21, 23]. Indeed, recent studies consistently show that postoperative complications are more closely associated with objective measures of patient frailty, nutritional status, disease severity and operative complexity, rather than demographic variables or disease onset timing [24–26].

This gap in risk stratification is not filled by existing tools. The American College of Surgeons National Surgical Quality Improvement Program (ACS NSQIP) Surgical Risk Calculator, though validated for general surgery, demonstrates insufficient accuracy for inflammatory bowel disease, generally underestimating risks and yielding modest discriminative performance [27]. Similarly, existing prognostic models for Crohn’s disease have primarily focused on predicting disease-related surgery or long-term complications, such as endoscopic recurrence, rather than perioperative surgical outcomes [28]. While several investigators have attempted to develop risk stratification tools incorporating clinical variables, inflammatory markers and disease characteristics, these models lack widespread validation, standardization and clinical implementation, representing a significant gap in perioperative care [29, 30].

The absence of a unified, surgery-specific scoring system is a substantial barrier to personalized surgical decision-making and informed consent. As the surgical management of Crohn’s disease becomes increasingly complex, with considerations for preoperative optimization, nutritional status, immunosuppressive therapy and anatomical complexity, there is an urgent imperative for the surgical community to develop and validate comprehensive, disease-specific risk assessment tools. Such tools must integrate both traditional surgical risk factors and Crohn’s-specific variables (e.g., disease duration, phenotype, medication exposure, nutritional parameters and inflammatory markers) to provide clinically actionable risk estimates. The development of such a standardized system, validated across diverse populations, represents a critical next step in advancing inflammatory bowel disease surgery and will be a primary focus of our future research.

This study’s findings should be interpreted within the context of its limitations. The primary weaknesses are its retrospective and single-centre design. Although robust statistical adjustment methods, including propensity score matching and G-computation, were employed to control for known confounders, the possibility of residual confounding from unmeasured variables cannot be entirely excluded. Furthermore, this patient cohort was drawn from a tertiary care referral centre. While this provided a sizeable and diverse patient group, it may limit the generalizability of our results to other settings, such as community hospitals or centres with different patient demographics and care models. Finally, while we stratified patients over 40 years (Montreal A3), our sample size did not permit a dedicated sub-analysis of the elderly population (> 60 years). It is possible that this specific subgroup exhibits a distinct, more indolent disease course that warrants focused investigation in future studies.

## Conclusions

This study found that age at diagnosis, as categorized by both the Vienna and Montreal classifications, does not predict short term surgical outcomes after ileocecal resection for Crohn’s disease. While younger patients present with distinct clinical phenotypes, these differences did not translate into higher postoperative morbidity. Instead, traditional markers of patient frailty, such as ASA score and comorbidity burden, were the dominant predictors of surgical risk, according to both regression and Random Forest analyses.

This highlights a critical, unmet need within the surgical community to develop a standardized, validated risk score specifically for Crohn’s disease surgery. The development of such a score, moving beyond traditional classifications to better quantify individual patient risk, will be the next goal of our research.

## Supplementary Information

Below is the link to the electronic supplementary material.

Supplementary file1 (DOCX 511 KB)

## Data Availability

No datasets were generated or analysed during the current study.
