# Supplementary material for: Rethinking risk in Crohn’s surgery: age at onset fails to predict surgical outcomes after ileocecal resection, insights from a tertiary referral center
Source: Tech Coloproctol. 2026 Apr 22;30(1):73. doi: 10.1007/s10151-026-03304-w (PMC13243319; doi:10.1007/s10151-026-03304-w)

| Operative time | Beta | p-val | LL | UL |
| --- | --- | --- | --- | --- |
| Raw | 2.837 | 0.557 | -6.630 | 12.304 |
| Multi | 4.394 | 0.341 | -4.652 | 13.440 |
| g-comp | 4.394 | 0.177 | -5.180 | 13.586 |
| PSM | 5.150 | 0.397 | -6.786 | 17.086 |
| 30-day post operative complication | OR | p-val | LL | UL |
| Raw | 1.320 | 0.084 | 0.962 | 1.809 |
| Multi | 1.213 | 0.300 | 0.841 | 1.747 |
| g-comp | 1.206 | 0.287 | 0.854 | 1.704 |
| PSM | 1.185 | 0.384 | 0.809 | 1.737 |
| 30-day readmission | OR | p-val | LL | UL |
| Raw | 0.637 | 0.262 | 0.270 | 1.335 |
| Multi | 0.602 | 0.276 | 0.227 | 1.434 |
| g-comp | 0.610 | 0.266 | 0.256 | 1.456 |
| PSM | 0.654 | 0.365 | 0.251 | 1.617 |
| Serious complications | OR | p-val | LL | UL |
| Raw | 1.513 | 0.108 | 0.903 | 2.487 |
| Multi | 1.451 | 0.231 | 0.781 | 2.647 |
| g-comp | 1.436 | 0.219 | 0.807 | 2.555 |
| PSM | 1.504 | 0.208 | 0.803 | 2.875 |
| 30-day reoperation | OR | p-val | LL | UL |
| Raw | 1.364 | 0.357 | 0.685 | 2.599 |
| Multi | 1.671 | 0.194 | 0.753 | 3.579 |
| g-comp | 1.653 | 0.181 | 0.792 | 3.451 |
| PSM | 1.593 | 0.290 | 0.682 | 3.910 |
| Length of hospital stay | OR | p-val | LL | UL |
| Raw | 2.048 | 0.000 | 1.079 | 3.017 |
| Multi | 1.915 | 0.000 | 0.848 | 2.982 |
| g-comp | 1.915 | 0.000 | 0.881 | 3.071 |
| PSM | 2.145 | 0.002 | 0.825 | 3.466 |

Supplementary Table 1: Full Adjusted Associations of Age at Diagnosis (Vienna Classification) with Surgical Outcomes

Supplementary Table 2: Full Adjusted Associations of Age at Diagnosis (Montreal Classification) with Surgical Outcomes.

| Operative time | β from A1 to A2 | | p-value | LL | UL | β from A1 to A3 | p-value | LL | UL |
| --- | --- | --- | --- | --- | --- | --- | --- | --- | --- |
| Raw | 9.726 | 0.144 | | -3.343 | 22.795 | 10.038 | 0.174 | -4.450 | 24.526 |
| Multi | 12.480 | 0.032 | | 1.059 | 23.901 | 14.028 | 0.042 | 0.503 | 27.553 |
| g-comp | 12.480 | 0.017 | | 0.536 | 23.835 | 14.028 | 0.023 | 0.218 | 27.733 |
| PSM | 10.251 | 0.043 | | 0.327 | 20.175 | 10.837 | 0.049 | 0.064 | 21.610 |
| Postop compl | OR A2 vs A1 | p-value | | LL | UL | OR A3 vs A1 | p-value | LL | UL |
| Raw | 0.724 | 0.147 | | 0.470 | 1.124 | 1.003 | 0.989 | 0.622 | 1.625 |
| Multi | 0.734 | 0.184 | | 0.466 | 1.163 | 0.916 | 0.751 | 0.534 | 1.576 |
| g-comp | 0.740 | 0.076 | | 0.465 | 1.179 | 0.919 | 0.385 | 0.544 | 1.662 |
| PSM | 0.789 | 0.173 | | 0.561 | 1.109 | 0.906 | 0.588 | 0.633 | 1.295 |
| Readmission | OR 2 vs 1 | p-value | | LL | UL | OR 3 vs 1 | p-value | LL | UL |
| Raw | 0.740 | 0.494 | | 0.328 | 1.893 | 0.529 | 0.232 | 0.185 | 1.550 |
| Multi | 0.891 | 0.808 | | 0.368 | 2.429 | 0.584 | 0.379 | 0.175 | 1.986 |
| g-comp | 0.894 | 0.500 | | 0.395 | 2.922 | 0.593 | 0.500 | 0.167 | 2.147 |
| PSM | 1.168 | 0.687 | | 0.551 | 2.542 | 0.885 | 0.777 | 0.371 | 2.069 |
| Srs-compl | OR 2 vs 1 | p-val | | LL | UL | OR 3 vs 1 | p-val | LL | UL |
| Raw | 0.433 | 0.011 | | 0.231 | 0.849 | 0.736 | 0.386 | 0.371 | 1.500 |
| Multi | 0.466 | 0.029 | | 0.239 | 0.949 | 0.690 | 0.377 | 0.304 | 1.594 |
| g-comp | 0.476 | 0.003 | | 0.246 | 0.996 | 0.697 | 0.159 | 0.331 | 1.535 |
| PSM | 0.548 | 0.036 | | 0.308 | 0.956 | 0.619 | 0.109 | 0.340 | 1.103 |
| Reop | OR 2 vs 1 | p-value | | LL | UL | OR 3 vs 1 | p-value | LL | UL |
| Raw | 0.262 | 0.001 | | 0.122 | 0.580 | 0.487 | 0.087 | 0.213 | 1.123 |
| Multi | 0.281 | 0.003 | | 0.124 | 0.655 | 0.562 | 0.250 | 0.209 | 1.516 |
| g-comp | 0.287 | 0.000 | | 0.123 | 0.687 | 0.568 | 0.101 | 0.228 | 1.496 |
| PSM | 0.361 | 0.006 | | 0.168 | 0.732 | 0.482 | 0.047 | 0.227 | 0.969 |
| LOS | β from A1 to A2 | p-value | | LL | UL | β from A1 to A3 | p-val | LL | UL |
| Raw | -1.045 | 0.126 | | -2.385 | 0.294 | 1.052 | 0.165 | -0.433 | 2.538 |
| Multi | -1.014 | 0.141 | | -2.365 | 0.337 | 0.872 | 0.285 | -0.729 | 2.473 |
| g-comp | -1.014 | 0.078 | | -2.532 | 0.273 | 0.872 | 0.159 | -0.992 | 2.480 |
| PSM | -0.819 | 0.126 | | -1.869 | 0.232 | 0.570 | 0.313 | -0.539 | 1.679 |

**Vienna Classification**

Supplementary Figure 1. 30-day postoperative complications

ASA

Comorbidities

Surgical approach

Conversion to open

Steroids at surgery

Additional procedures

Vienna Classification

Years of disease

Previous use of biologics

BMI

Gender


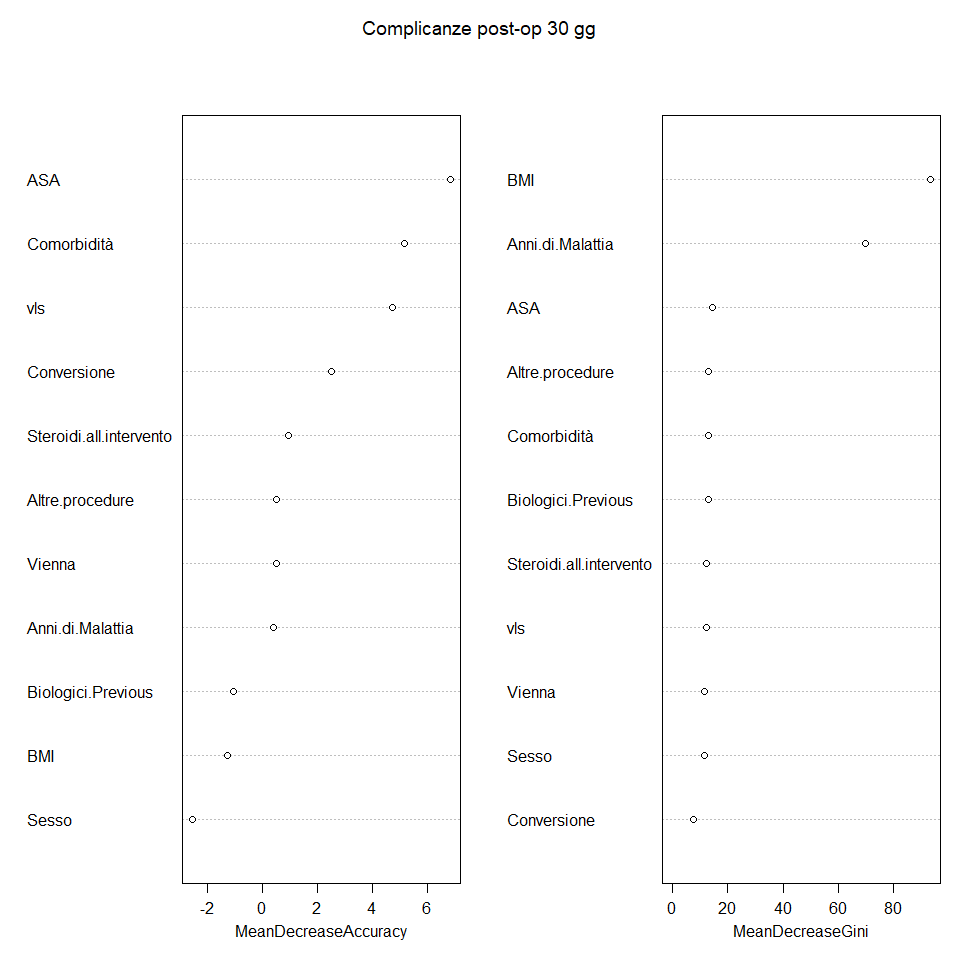


Supplementary Figure 2. 30-day reoperation

ASA

Conversion to open

BMI

Additional procedures

Surgical approach

Comorbidities

Vienna Classification

Gender

Previous use of biologics

Years of disease

Steroids at surgery


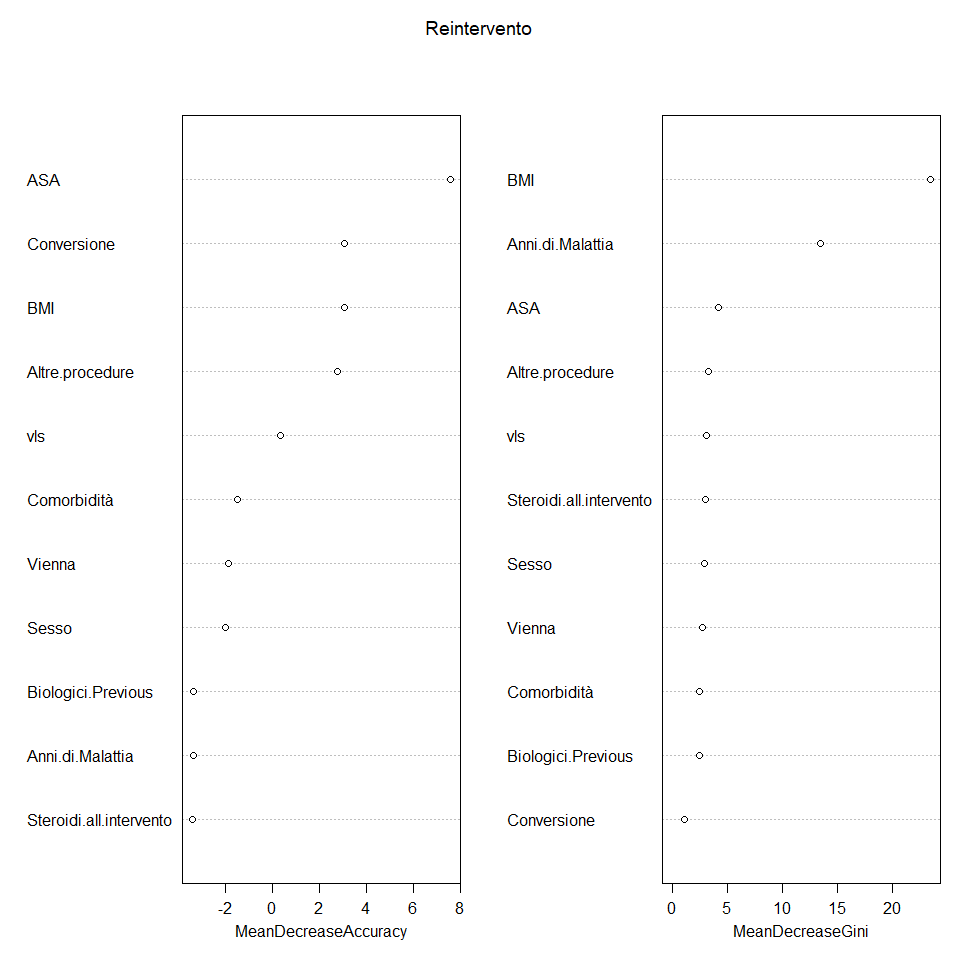


Supplementary Figure 3. 30-day postoperative serious complications

ASA

BMI

Comorbidities

Additional procedures

Surgical approach

Vienna Classification

Gender

Steroids at surgery

Previous use of biologics

Conversion to open

Years of disease


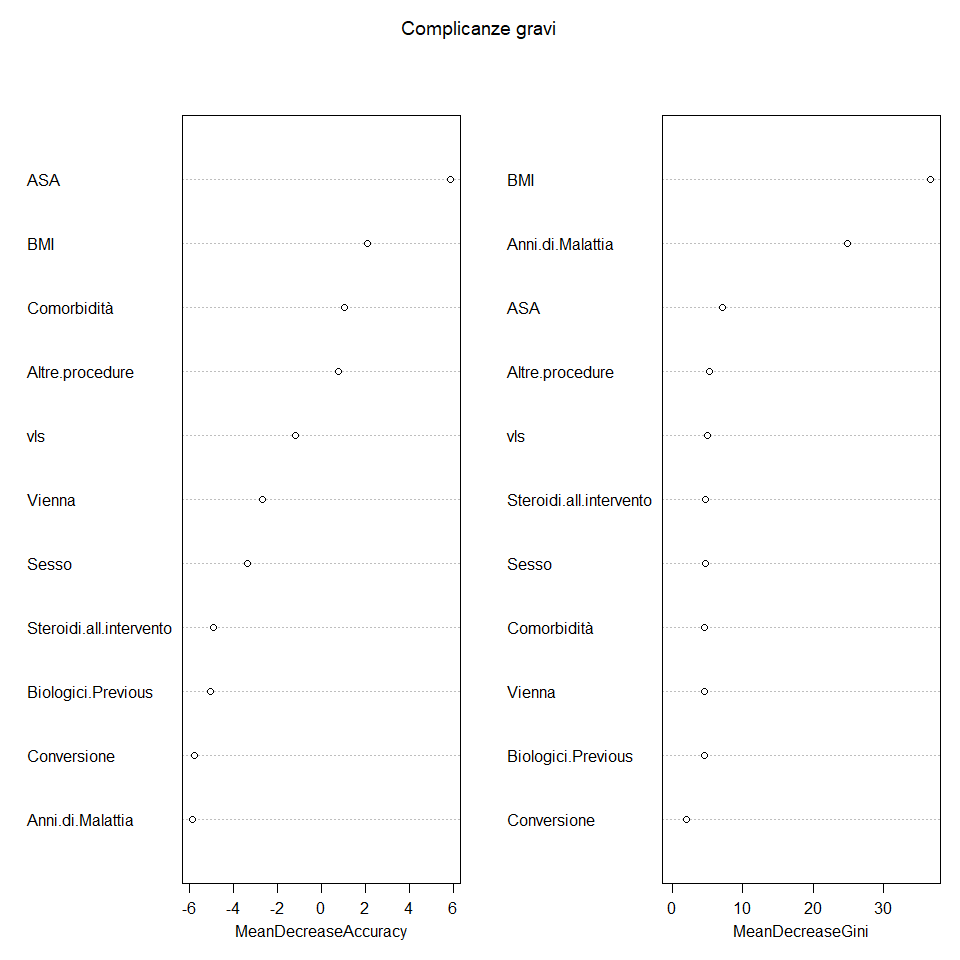


Supplementary Figure 4. Operative time

Additional procedures

Conversion to open

Years of disease

BMI

Surgical approach

Gender

Vienna Classification

Comorbidities

Previous use of biologics

ASA

Steroids at surgery


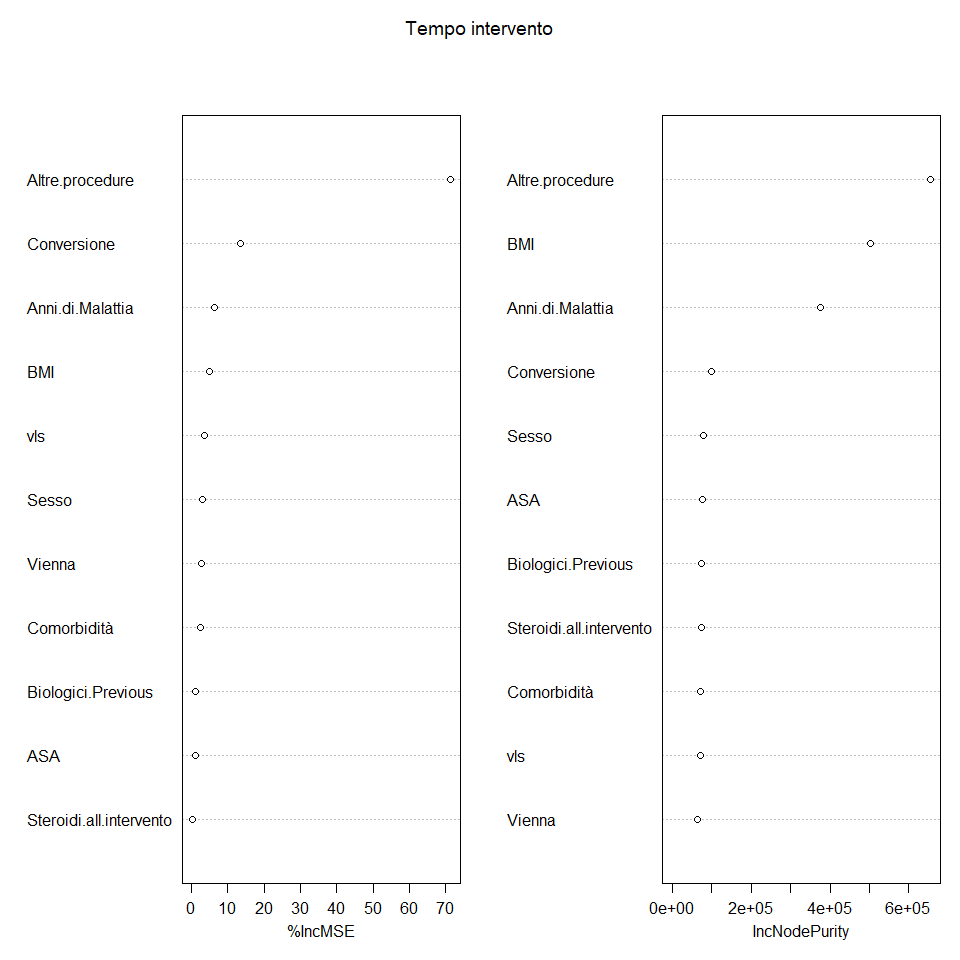


Supplementary Figure 5. Length of stay

Surgical approach

Comorbidities

ASA

BMI

Vienna Classification

Gender

Conversion to open

Steroids at surgery

Additional procedures

Previous use of biologics

Years of disease


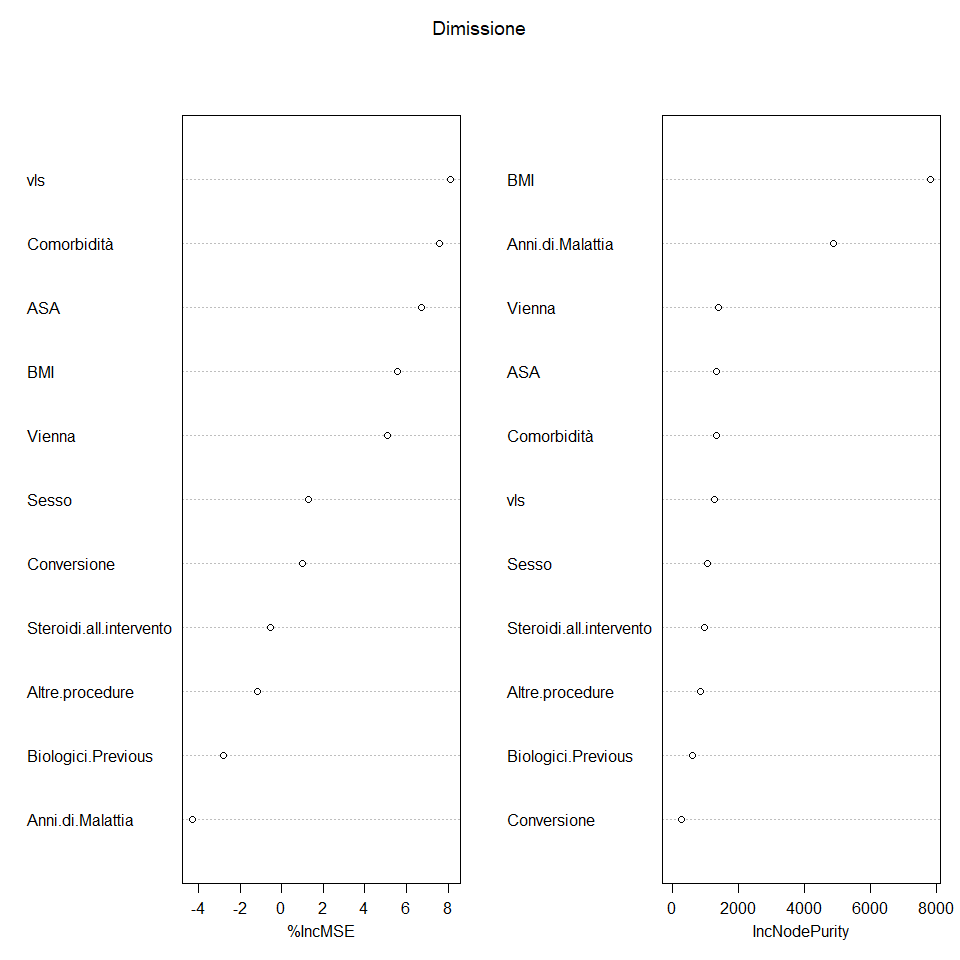


**Montreal Classification**

Supplementary Figure 6. 30-day postoperative complications

ASA

Surgical approach

Comorbidities

Montreal Classification

Conversion to open

Steroids at surgery

BMI

Years of disease

Additional procedures

Previous use of biologics

Gender


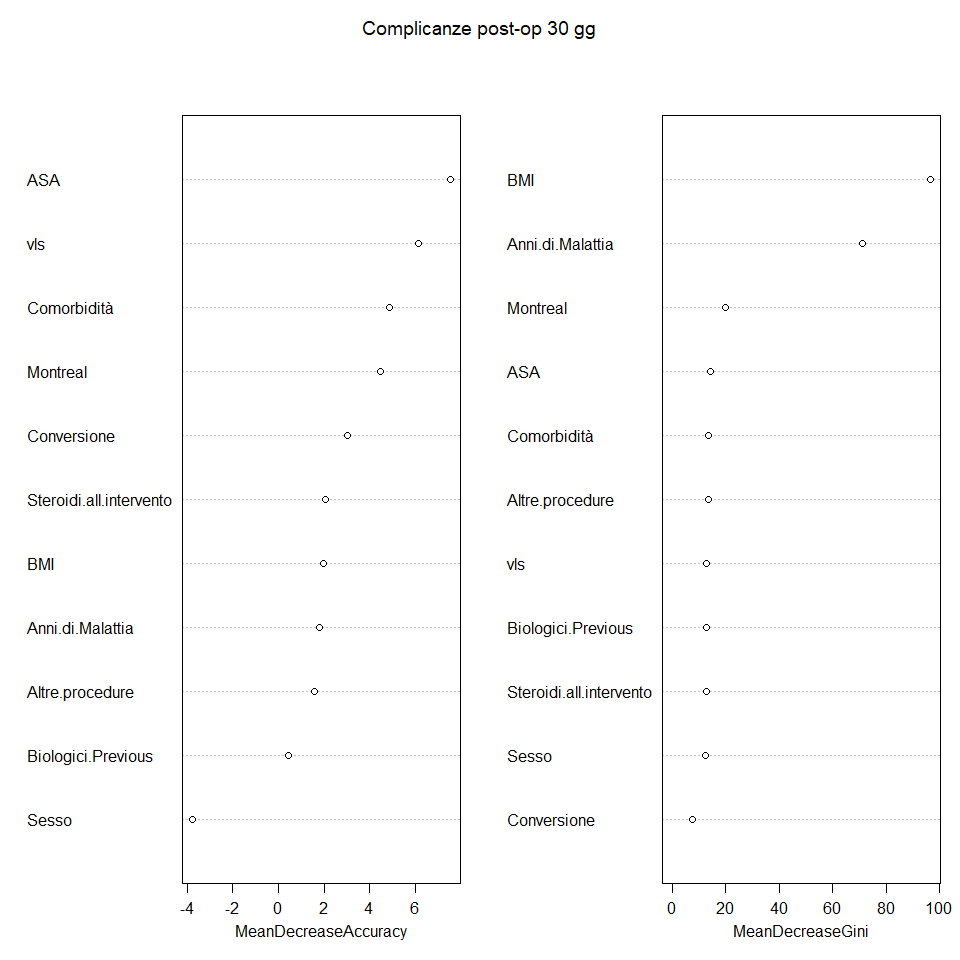


Supplementary Figure 7. 30-day reoperation

ASA

Additional procedures

BMI

Conversion to open

Comorbidities

Montreal Classification

Years of disease

Surgical approach

Steroids at surgery

Gender

Previous use of biologics


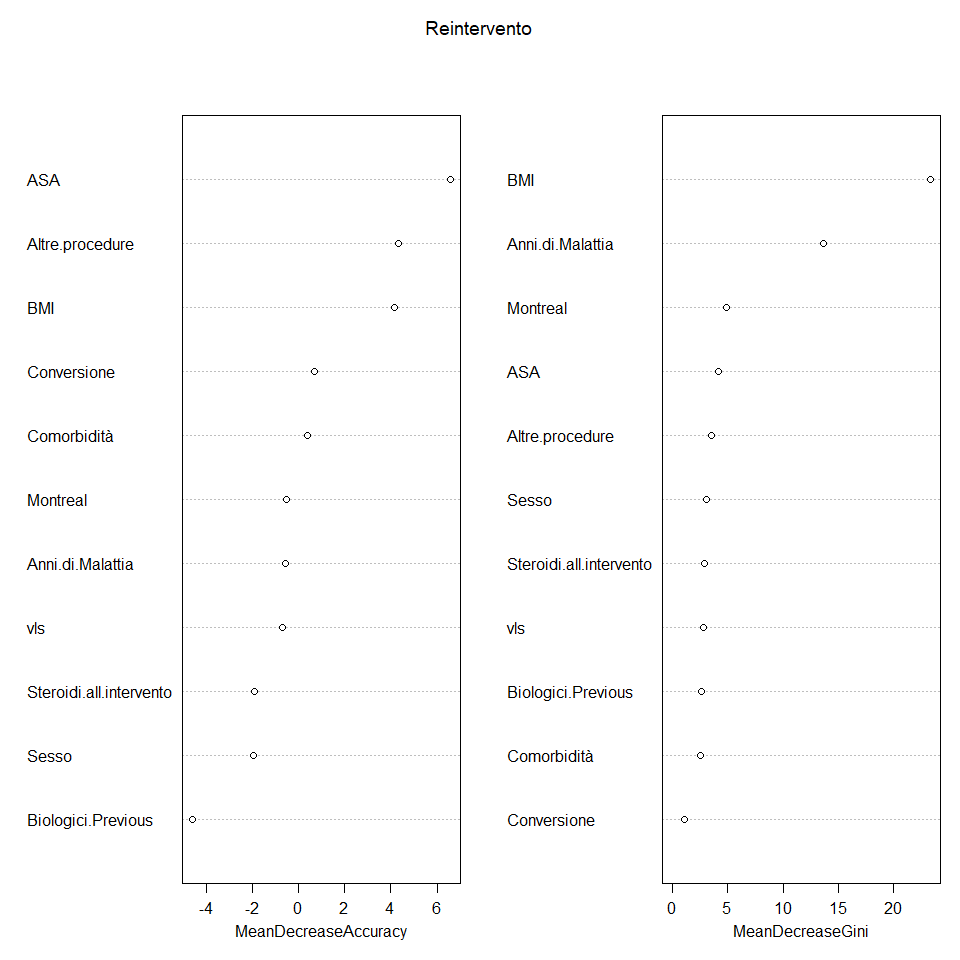


Supplementary Figure 8. 30-day postoperative serious complications

ASA

BMI

Conversion to open

Comorbidities

Additional procedures

Surgical approach

Montreal Classification

Gender

Years of disease

Previous use of biologics

Steroids at surgery


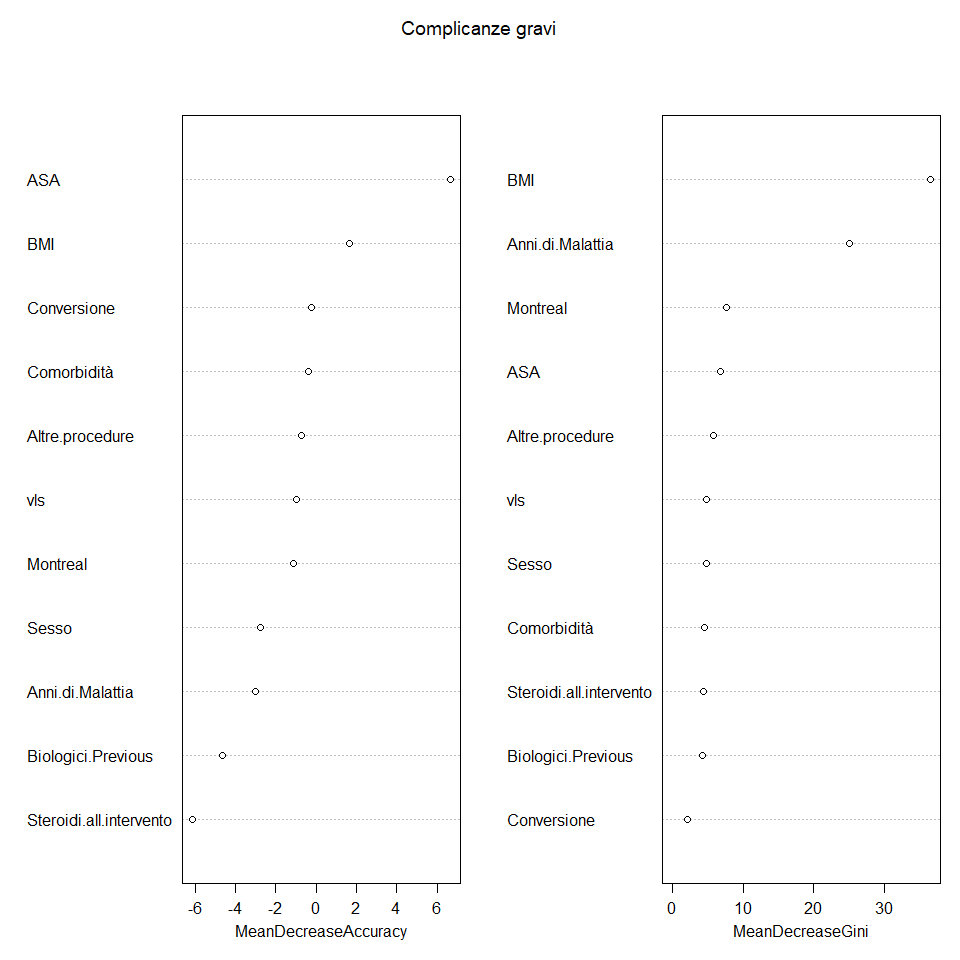


Supplementary Figure 9. Operative time

Additional procedures

Conversion to open

Years of disease

BMI

Previous use of biologics

Gender

Comorbidities

Surgical approach

Montreal Classification

ASA

Steroids at surgery


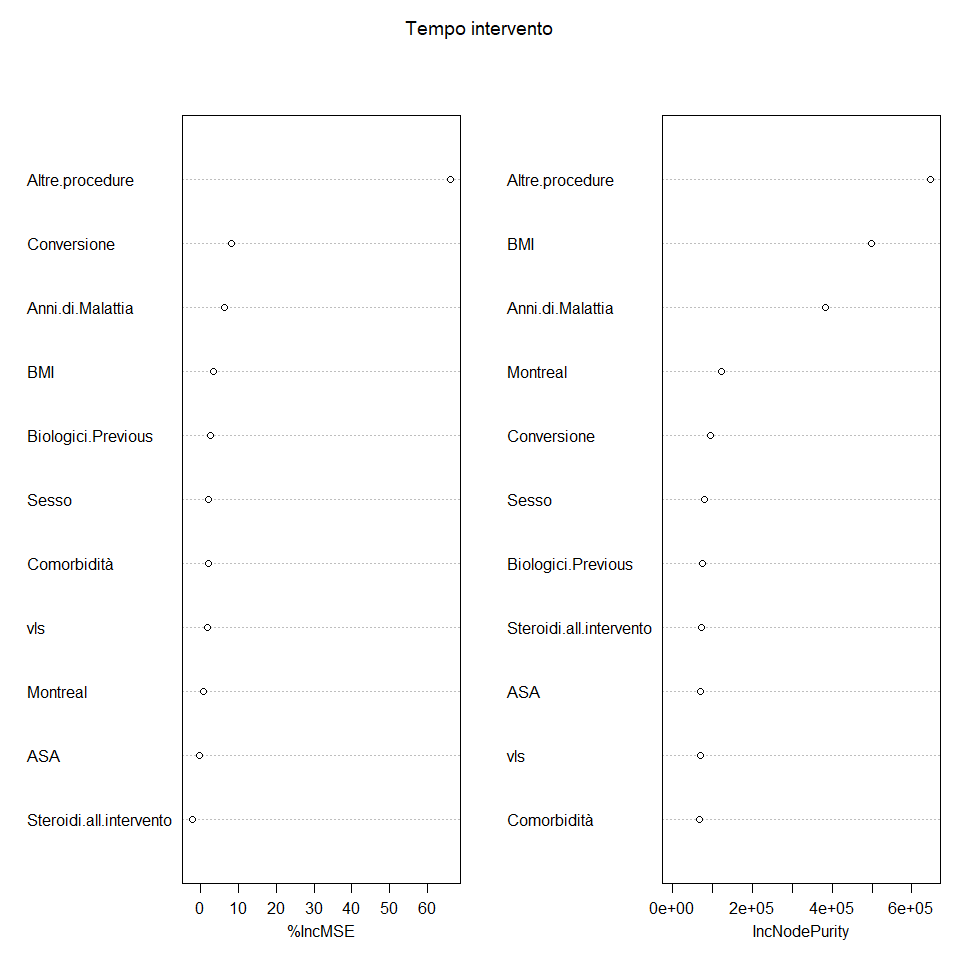


Supplementary Figure 10. Length of stay

ASA

Comorbidities

Surgical approach

Montreal Classification

Gender

BMI

Steroids at surgery

Conversion to open

Additional procedures

Years of disease

Previous use of biologics


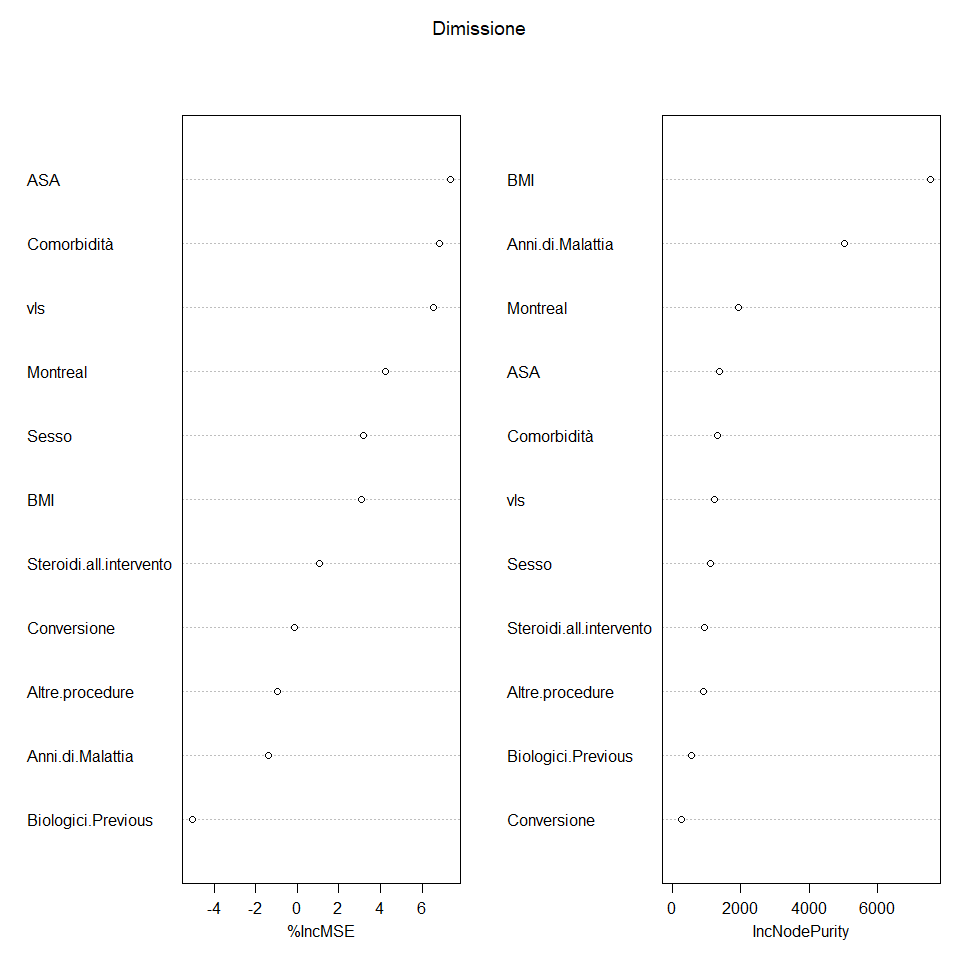

Supplement: Supplementary file 1 — Supplementary file1 (DOCX 511 KB) [file 10151_2026_3304_MOESM1_ESM.docx]
